# Shorter vs. standard-duration antibiotic therapy for nocardiosis: a multi-center retrospective cohort study

**DOI:** 10.1007/s15010-024-02445-0

**Published:** 2024-11-26

**Authors:** Nofar Hezkelo Attias, Tal Schlaeffer-Yosef, Itay Zahavi, Noga Hasson, Yaara Ben Ari, Basel Darawsha, Idan Levitan, Elad Goldberg, Michal Landes, Vladislav Litchevsky, Haim Ben-Zvi, Sharon Amit, Lior Nesher, Jihad Bishara, Mical Paul, Dafna Yahav, Ili Margalit

**Affiliations:** 1 Internal Medicine F, Rabin Medical Center, Beilinson Hospital, Petah-Tikva, Israel; 2 Infectious Diseases Institute, Soroka Medical Center, Beer Sheba, Israel; 3 Faculty of Health Sciences, Ben-Gurion University of the Negev, Beer Sheba, Israel; 4 The Ruth and Bruce Rappaport Faculty of Medicine, Technion-Israel Institute of Technology, Haifa, Israel; 5 School of Medicine, Faculty of Medical and Health Sciences, Tel Aviv University, Ramat Aviv, Tel Aviv, Israel; 6 Lev Hasharon Mental Health Center, Tzur Moshe, Israel; 7 Department of Neurosurgery, Rabin Medical Center, Beilinson Hospital, Petah-Tikva, Israel; 8 Internal Medicine D, Rabin Medical Center, Beilinson Hospital, Petah-Tikva, Israel; 9 Infectious Diseases Unit, Sheba Medical Center, Sheba Road 2, Ramat-Gan, Israel; 10 Microbiology Laboratory, Rabin Medical Center, Beilinson Hospital, Petah-Tikva, Israel; 11 Microbiology Laboratory, Sheba Medical Center, Ramat-Gan, Israel; 12 Infectious Diseases Unit, Rabin Medical Center, Beilinson Hospital, Petah-Tikva, Israel; 13 Infectious Diseases Institute, Rambam Healthcare Campus, Haifa, Israel

**Keywords:** Immune suppression, Nocardia, Opportunistic infections, Treatment

## Abstract

**Purpose:**

The prolonged treatment recommended for nocardiosis does not rely on strong evidence. Consequently, some clinicians opt shorter therapy in certain circumstances. We assessed the effectiveness of shorter therapy.

**Methods:**

A multi-center retrospective cohort study comprising individuals diagnosed with nocardiosis between 2007 and 2022. We classified all patients who survived 90 days into three groups according to treatment duration: short (≤ 90 days), intermediate (91–180 days), and prolonged (> 180 days). We compared baseline characteristics (comorbidities, immune status) and nocardiosis manifestations across the unadjusted treatment groups, one-year all-cause mortality, disease relapse, and antibiotic-related adverse events to identify patients who may safely receive the short course.

**Results:**

We detected 176 patients with nocardiosis, their median age was 65 years; 74 (42%) were women. Forty-three (24%) patients died within 90 days. Of the remaining 133, 37 (28%) patients received short therapy, 40 (30%) intermediate, and 56 (42%) prolonged treatment duration. Longer courses were more likely to be administered to patients with immunosuppression, disseminated nocardiosis, and *N. farcinica* infection. Within a year, 20 (15%) individuals died and 2 (2%) relapsed. Treatment duration was not associated with either mortality (*p* = 0.945) or relapse (*p* = 0.509). Nocardiosis was the cause of death in only one patient, receiving a prolonged course. Of 73 patients with solitary pulmonary nocardiosis, 20 (27%) received short duration. None relapsed and 2 (10%) died, both immunocompromised. The rate of AE was similar across the groups.

**Conclusions:**

With clinically guided case-by-case patient selection nocardiosis can be safely treated for durations significantly shorter than traditionally recommended.

**Supplementary Information:**

The online version contains supplementary material available at 10.1007/s15010-024-02445-0.

## Introduction

Nocardiosis is traditionally treated for long durations, ranging from 6 to 12 months. Some recommend a shorter duration (≤ 3 months) for immunocompetent individuals with cutaneous infection [1, 2]. This tendency for extended antibiotic courses is based on retrospective small case-series published over four decades ago when patients were usually treated with sulfonamides monotherapy or with trimethoprim-sulfamethoxazole (SXT) alone. In these circumstances, individuals who were treated for ≤ 3 months had higher relapse rates in comparison to those who completed longer treatment courses, usually for six months [3, 4].

Nowadays, pulmonary or disseminated nocardiosis is initially treated with combination regimens of ≥ 2 drugs (usually SXT or linezolid combined with another bactericidal agent). Consequently, preliminary evidence implies that treatment duration might be shorter than previously assumed. Among 12 heart transplant recipients with pulmonary nocardiosis who were treated for up to 120 days, 92% (11/12) were cured (the only death was unrelated to nocardiosis) [5]. A retrospective assessment of 107 solid organ transplant recipients (SOTRs) with nocardiosis who completed their scheduled treatment, found that the 17 SOTRs receiving short course (≤ 120 days) tended to have milder infectious syndromes. Their 1-year cure rate was high (88%, 15/17), with only one relapse and one mortality case (related to candidemia) [6]. Although evidence is scarce, clinicians administer shorter antibiotic courses in selected cases, and treatment durations of < 120 days are being administered even for substantially immunosuppressed individuals [7]. 

Randomized control trials (RCT) for nocardiosis are impractical, considering its relative rarity, heterogeneous manifestations, and diverse host characteristics. To address this unmet need in the era of shortening antibiotic courses for infections, we conducted this real-life study to assess the effectiveness of shorter treatment durations for nocardiosis.

## Methods

### Study design and setting

We conducted a multi-center retrospective cohort study of adults (aged ≥ 18 years) who were diagnosed with nocardiosis between 2007 and 2022.

The cohort comprised individuals from four tertiary medical centers in Israel: Rabin Medical Center– Beilinson Hospital, Sheba Medical Center, Rambam Healthcare Campus, and Soroka Medical Center. Characteristics of participating centers and periods of patients’ identification are provided in supplementary Table S1. We reviewed the microbiology laboratory records of participating centers and retrieved the medical charts of all individuals from whom Nocardia was isolated up to December 31st, 2022, allowing at least one-year follow-up for all included individuals.

### Eligibility criteria

We defined nocardiosis as Nocardia species isolation from an individual who received a formal diagnosis of nocardiosis by an infectious diseases specialist and was treated with at least one agent directed against Nocardia species, either empirically or as guided by antibiotic susceptibility testing. Nocardia colonization was defined as Nocardia isolation from an individual who had no relevant symptoms, was not diagnosed with nocardiosis, and did not receive treatment directed at Nocardia [8]. We included all individuals with nocardiosis and excluded all cases of Nocardia colonization. Individuals with nocardiosis for whom data on treatment duration were unavailable or could not be ascertained were also excluded.

### Exposure variables

Our primary exposure variable was the total antibiotic treatment duration, defined as the period in which treatment directed against nocardiosis was given, regardless of antibiotic type, number of agents, and route of administration, as these could change throughout treatment (e.g., switching from combination to monotherapy). Secondary prophylaxis with oral low-dose SXT (i.e., 800/160 mg once daily or less frequently, following a treatment period at therapeutic doses or other agents) was not counted as treatment.

Based on the previously suggested treatment algorithm [2], we classified the cohort into three treatment duration groups: short (≤ 90 days), intermediate (91–180 days), and prolonged (> 180 days). Intravenous treatment duration (defined as the period in which at least one agent was administered intravenously) and combination therapy duration (i.e., the period in which ≥ 2 agents were administered) were also calculated.

### Additional independent variables

We collected data on patients’ baseline characteristics, including demographics, comorbidities, and immune status [see below]. Information on nocardiosis included the causative Nocardia species, the infection syndrome [see below], and presentation (need for oxygen and markers of inflammation). Data on surgical interventions for either diagnostic workup or therapeutic purposes (debridement, drainage of an abscess, heart valve replacement, etc.) were also collected.

We classified patients into three immune status groups, based on their underlying comorbidities and pharmacotherapy (i.e., use of immunosuppressive or immunomodulatory agents), according to the level of immune suppression: substantial immune suppression (SOTRs, hematopoietic stem cell transplant recipients [HCTs], individuals with acquired immunodeficiency syndrome [AIDS], and those on high-dose corticosteroid therapy [≥ 20 mg prednisone equivalent per day] for at least 21 days prior to nocardiosis); mild to moderate immune suppression (individuals with active malignancy, primary immune deficiency, autoimmune diseases, usage of immunomodulatory agents and those on medium dose corticosteroid therapy [≥ 5 and < 20 mg prednisone equivalent per day] for at least 21 days) prior to nocardiosis; and apparently immunocompetence (all others).

Nocardiosis was classified into one of five syndromes: solitary lymphocutaneous nocardiosis (defined as an infection involving skin and soft tissue only); Solitary pulmonary nocardiosis (defined as an infection involving only the lungs’ parenchyma, with or without empyema); disseminated nocardiosis without central nervous system (CNS) involvement (defined as involvement of ≥ 2 non-contiguous organs or Nocardia bacteremia), disseminated nocardiosis with CNS involvement (defined as any infection involving the CNS), and Nocardia osteomyelitis.

### Outcome variables

Our primary outcome was 1-year all-cause mortality.

Secondary outcomes included nocardiosis relapse and antibiotic-related adverse events within one year.

Nocardiosis relapse was defined as the recurrence of signs and symptoms, potentially following resolution period, with repeated microbiological evidence of the same Nocardia species.

Antibiotic-related adverse events were treated as a composite outcome, including at least one of the following: renal toxicity, bone marrow toxicity, *Clostridioides Difficile* infection (CDI), and allergic reactions related to the regimen used against nocardiosis (definitions for each of the four adverse events are provided in the supplementary material (supplementary Table S2).

### Data sources and management

Information on the study variables was manually extracted from patients’ medical charts. We included all hospital visits (either admissions or outpatient clinics) during the year following the diagnosis and recorded all antibiotic therapy, dates of regimen alternations (in either agent, dosage, or route of administration), dates of treatment secession, and microbiology (e.g., repeated cultures).

Data collected through different sources (hospital and community records) were cross-checked against each other to guarantee their consistency and reliability.

### Laboratory methods

Sputum, bronchoalveolar lavage, blood, and tissue samples from infected individuals were incubated on blood and chocolate agar plates for at least 2–4 days. When Nocardia was suspected, samples were seeded on additional plates (such as Tayer–Martin agar or Buffered Charcoal Yeast Extract) and incubated for longer durations.

Nocardia identification was based on macroscopic and microscopic assessment combined with biochemical markers early during the study years and matrix-assisted laser desorption/ionization time-of-flight mass spectrometer (MALDI-TOF MS) or sequencing when it became available. Whenever performed, antimicrobial susceptibility testing was performed using either broth microdilution or E-testing (supplementary Table S3).

### Statistical analysis

Treatment duration was cross-tabulated with immune status, nocardiosis syndromes, Nocardia species, and other demographic and clinical characteristics. Treatment duration groups were compared using Chi square or Fisher’s exact tests for categorical variables and one-way ANOVA or the Mann-Whitney-U test for continuous variables. Kaplan–Meier survival curves were plotted and assessed for the primary outcome using the Log-rank test.

Considering the immense heterogeneity in clinical syndromes and host-pathogen relationships, making nocardiosis cases non-comparable by no means, we decided to renounce adjustments, recognizing that clinical judgment has led clinicians to shorten treatment durations in appropriate cases. Accordingly, multivariable analysis was not conducted.

To validate our findings, we implemented several sensitivity analyses:

Considering that we excluded individuals who died during the 90 days from nocardiosis diagnosis while on treatment, we reanalyzed the data following an exclusion of all individuals who died while on therapy, at any time point (i.e., even beyond 90 days).

Additionally, we reanalyzed our data following reclassification of the treatment duration groups, once by including those who were treated for 91–120 days in the short treatment group, and once by combining the short and intermediate groups (i.e., short-intermediate vs. prolonged treatment duration, using a single cutoff of 180 days).

For all analyses, *p* < 0.05 was considered statistically significant.

We analyzed data using IBM SPSS version 28 (Armonk, New York, USA) and GraphPad Prism 8.0.1.

### Ethical considerations

The study was approved by the Institutional Review Boards (IRB) of each participating center (RMC-0685-21; SMC-9959-22; RMB-D-0039-23; SOR-0046-23). The IRBs granted an exemption from obtaining informed consent due to the study’s retrospective design, and we followed the STROBE guidelines for observational studies [9]. 

## Results

During the study period, Nocardia was isolated from 245 individuals, of whom medical charts were available for 241 (98%). Forty-six (19%) individuals identified as having Nocardia colonization were excluded. Of the remaining 195 individuals with nocardiosis, 19 (10%) were excluded since their treatment duration could not be ascertained. The remaining 176 individuals had a median age of 65 (IQR 23; range 18–92) years and 74 (42%) were women. By one year from diagnosis, 63 (36%) individuals have died, 43 (68%) of them within 90 days from diagnosis. The latter died while on anti-nocardial therapy, among 16 (37%) out of them, nocardiosis was deemed as the cause of death (supplementary Table S4). further excluded, leaving 133 individuals with nocardiosis for treatment duration analysis. These were classified into three groups: short (*N* = 37, 28%), intermediate (*N* = 40, 30%), and prolonged (*N* = 56, 42%) treatment duration.

**Fig. 1:**
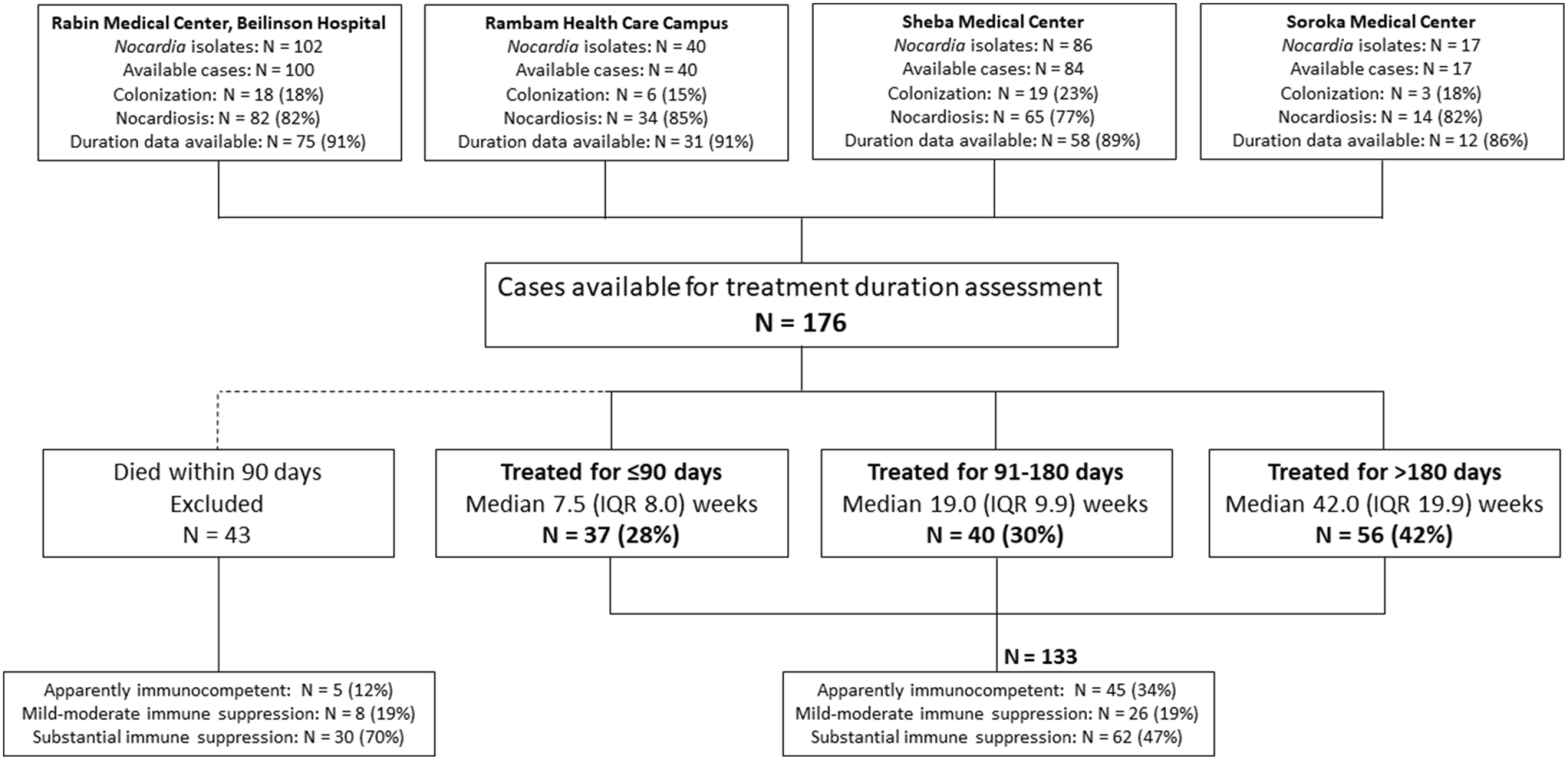
Study flow diagram

Two-thirds of the cohort were classified as having any type of immune suppression (26/133 [19%] and 62/133 [47%] with mild-moderate and substantial immune suppression, respectively), while one-third was deemed immunocompetent (see Fig. 1 for study flow diagram).

**Fig. 2:**
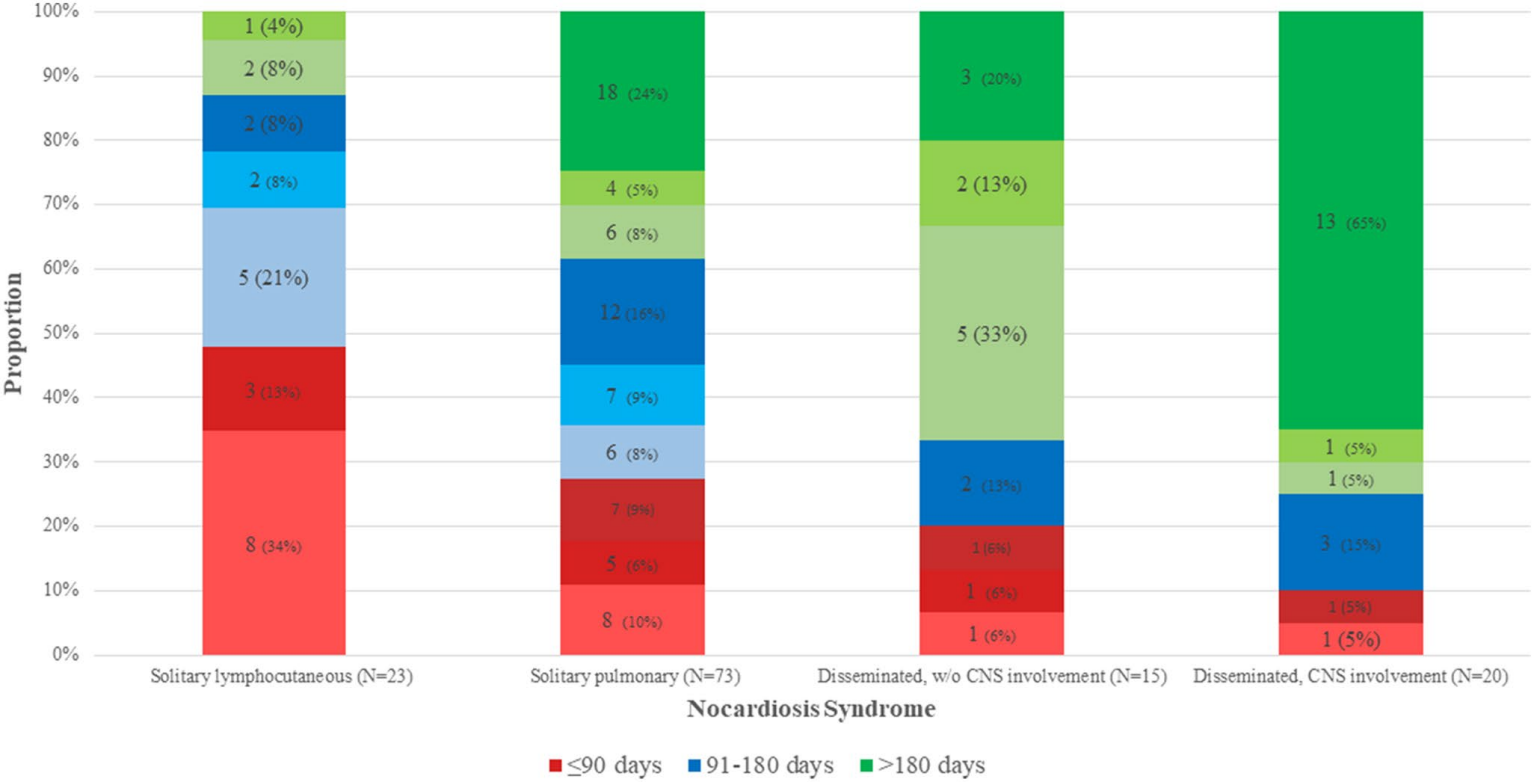
Distribution of treatment duration groups according to nocardiosis clinical syndrome and immune status. For each treatment duration group (represented by colors), the shades signify the immune status (apparent immunocompetence, and mild-moderate or substantial immune suppression): the darker the shade the diminished the immunity

Most individuals 111/133 (84%) were treated with SXT-based regimens, regardless of treatment duration (*p* = 0.617). However, therapy group positively correlated with the proportion of individuals receiving combination regimens (43%, 70%, and 82% for short, intermediate, and prolonged treatment duration, respectively, *p* < 0.001), the proportion of intravenous antibiotics administration (6 [IQR 8], 6 [10], 14 [26] weeks for short, intermediate, and prolonged treatment duration, respectively, *p* < 0.001), and with longer duration of intravenous antibiotics (1 [IQR 6], 3 [12], and 6 [22] weeks, for short, intermediate, and prolonged treatment duration, respectively, *p* < 0.001).

### Correlates of treatment duration

Patients’ immunity was the only baseline characteristic correlated with treatment duration groups. Higher proportions of individuals with substantial immune suppression (24%, 48%, and 61% for short, intermediate, and prolonged treatment duration, respectively, *p* = 0.014) and recent use of systemic corticosteroids (30%, 58%, and 61% for short, intermediate, and prolonged treatment duration, respectively, *p* = 0.009) were observed in the more extended duration groups. Respectively, 19/37 (51%) of those receiving short treatment duration were immunocompetent (Table 1).

**Table 1:** Clinical characteristics and outcomes of individuals diagnosed with nocardiosis, according to treatment duration

	Died within 90 days *N* = 43 (24%)	Treatment duration			*p* ^a^
		≤ 90 days *N* = 37 (28%)	91–180 days *N* = 40 (30%)	> 180 days *N* = 56 (42%)	
**Demographics and comorbidities**					
Age at diagnosis, median (IQR)	67 (21)	56 (30)	67 (25)	61 (22)	0.352
Female gender, N (%)	14 (32.6)	17 (45.9)	17 (42.5)	26 (46.4)	0.923
Charlson score, median (IQR)	4 (4)	2.0 (3.0)	2.0 (3.0)	2.0 (3.0)	0.420
Solid organ transplant recipient, N (%)	4 (9.3)	2 (5.4)	8 (20.0)	10 (17.9)	0.149
Hematopoietic stem cell transplant recipient, N (%)	5 (11.6)	1 (2.7)	4 (10.0)	9 (16.1)	0.122^*^
Chronic pulmonary disease, N (%)	14 (32.6)	13 (35.1)	18 (45.0)	25 (44.6)	0.600
Malignancy, N (%)	26 (60.5)	8 (21.6)	10 (25.0)	17 (30.4)	0.629
Autoimmune disease, N (%)	8 (18.6)	5 (13.5)	7 (17.5)	14 (25.0)	0.364
Primary immune deficiency, N (%)	0	2 (5.4)	2 (5.0)	0	0.173^*^
Diabetes mellitus, N (%)	18 (41.9)	11 (29.7)	12 (30.0)	21 (37.5)	0.653
Systemic corticosteroid therapy, N (%)	32 (74.4)	11 (29.7)	23 (57.5)	34 (60.7)	**0.009**
Prednisone equivalent dose, median (IQR) ^b^	27 (32)	30 (35)	20 (33)	28 (50)	0.253
Prednisone > 20 mg per days, N (%)	28 (65.1)	7 (18.9)	13 (32.5)	24 (42.9)	0.056
Immune status					**0.014**
Apparently immunocompetent, N (%)	5 (11.6)	19 (51.4)	12 (30.0)	14 (25.0)	
Mild-moderate immune suppression ^c^, N (%)	8 (18.6)	9 (24.3)	9 (22.5)	8 (14.3)	
Substantial immune suppression ^d^, N (%)	30 (69.8)	9 (24.3)	19 (47.5)	34 (60.7)	
**Nocardiosis**					
*Nocardia* species ^e^					0.057^*^
*Nocardia cyriacigeorgica*, N (%)	11 (45.8)	10 (50.0)	13 (61.9)	13 (43.3)	
*Nocardia farcinica*, N (%)	7 (29.2)	1 (5.0)	3 (14.3)	8 (26.7)	
*Nocardia otitidiscaviarum*, N (%)	3 (12.5)	0	1 (4.8)	3 (10.0)	
*Nocardia brasiliensis*, N (%)	0	5 (25.0)	1 (4.8)	0	
Miscellaneous *Nocardia* spp., N (%)	3 (12.5)	4 (20.0)	3 (14.3)	6 (20.0)	
Nocardiosis syndrome					**0.001** ^*^
Solitary lymphocutaneous, N (%)	0	11 (29.7)	9 (22.5)	3 (5.4)	
Osteomyelitis, N (%)	1 (2.3)	1 (2.7)	1 (2.5)	0	
Solitary pulmonary, N (%)	24 (55.8)	20 (54.1)	25 (62.5)	28 (50.0)	
Disseminated without CNS involvement, N (%)	3 (7.0)	3 (8.1)	2 (5.0)	10 (17.9)	
Disseminated with CNS involvement, N (%)	15 (34.9)	2 (5.4)	3 (7.5)	15 (26.8)	
Oxygen supplementation during hospitalization					0.624^*^
No need, N (%)	10 (23.8)	23 (74.2)	25 (73.5)	35 (63.6)	
Nasal canula, N (%)	10 (23.8)	3 (9.7)	6 (17.6)	11 (20.0)	
High-flow nasal canula, N (%)	4 (9.5)	1 (3.2)	0	4 (7.3)	
Non-invasive ventilation, N (%)	2 (4.8)	0	0	0	
Mechanical ventilation, N (%)	16 (38.1)	4 (12.9)	3 (8.8)	5 (9.1)	
C-reactive protein at presentation (mg/dL), median (IQR)	12.6 (22.0)	1.9 (17.0)	8.1 (12.0)	9.1 (18.0)	0.284
**Treatment**					
SXT-based regimen, N (%)	37 (86.0)	29 (78.4)	34 (85.0)	48 (85.7)	0.617
Combination treatment, N (%)	33 (76.7)	16 (43.2)	28 (70.0)	46 (82.1)	**< 0.001**
Combination treatment duration (weeks), median (IQR)	2.0 (5.0)	6.3 (7.8)	6.0 (10.4)	13.5 (26.0)	**< 0.001**
IV therapy duration (weeks), median (IQR)	1.8 (2.9)	0.8 (5.5)	3.0 (11.8)	5.5 (21.6)	**< 0.001**
Antibiotic-related side effects, N (%)	16 (37.2)	12 (32.4)	18 (45.0)	19 (33.9)	0.437
Renal toxicity, N (%)	6 (15.0)	2 (5.7)	6 (17.6)	8 (14.3)	0.283^*^
Bone marrow toxicity, N (%)	9 (22.5)	5 (14.3)	7 (19.4)	7 (12.7)	0.674
*C. difficile* infection, N (%)	1 (2.5)	2 (5.7)	1 (2.9)	3 (5.4)	0.999^*^
Allergic reaction, N (%)	0	3 (8.6)	4 (11.4)	5 (9.1)	0.930^*^
Surgical intervention, N (%)	4 (9.8)	8 (22.2)	8 (21.6)	14 (25.5)	0.895
**Outcomes at one year from diagnosis**					
Rehospitalization, N (%)	NA	17 (45.9)	18 (45.0)	29 (51.8)	0.768
Nocardiosis relapse, N (%)	NA	0	0	2 (3.6)	0.509^*^
All-cause mortality, N (%)	NA	5 (13.5)	6 (15.0)	9 (16.1)	0.945

^a^ Calculated for the three treatment groups using Chi-square test or Fisher’s exact test (^*^) and one-way ANOVA for categorical and continuous variables, respectively; ^b^ Average daily prednisone dosage (in milligrams), during the 90 days prior to presentation. Whenever dexamethasone was used, dosage was converted to the equivalent prednisone dosage; ^c^ Individuals with active malignancy, primary immune deficiency, autoimmune diseases, and chronic corticosteroid therapy (≥ 5 and < 20 mg per day); ^d^ Solid organ transplant recipients, hematopoietic stem cell transplant recipients, and individuals with acquired immunodeficiency syndrome (AIDS) or on chronic corticosteroid therapy (≥ 20 mg per day); ^e^ Identification to the species level was available for 95/176 (54%) of the cohort. IV = intravenous; NA = not applicable; SXT = sulfamethoxazole-trimethoprim

Nocardiosis manifestations (i.e., clinical syndromes) associated with treatment duration (*p* = 0.001). Individuals with solitary lymphocutaneous nocardiosis received shorter treatment durations while individuals with disseminated nocardiosis were administered with longer durations (Fig. 2).

Of those for whom Nocardia was identified to the species level, 75% (8/12) with *N. farcinica* received a prolonged treatment course (Table 1).

### Outcomes

**Fig. 3:**
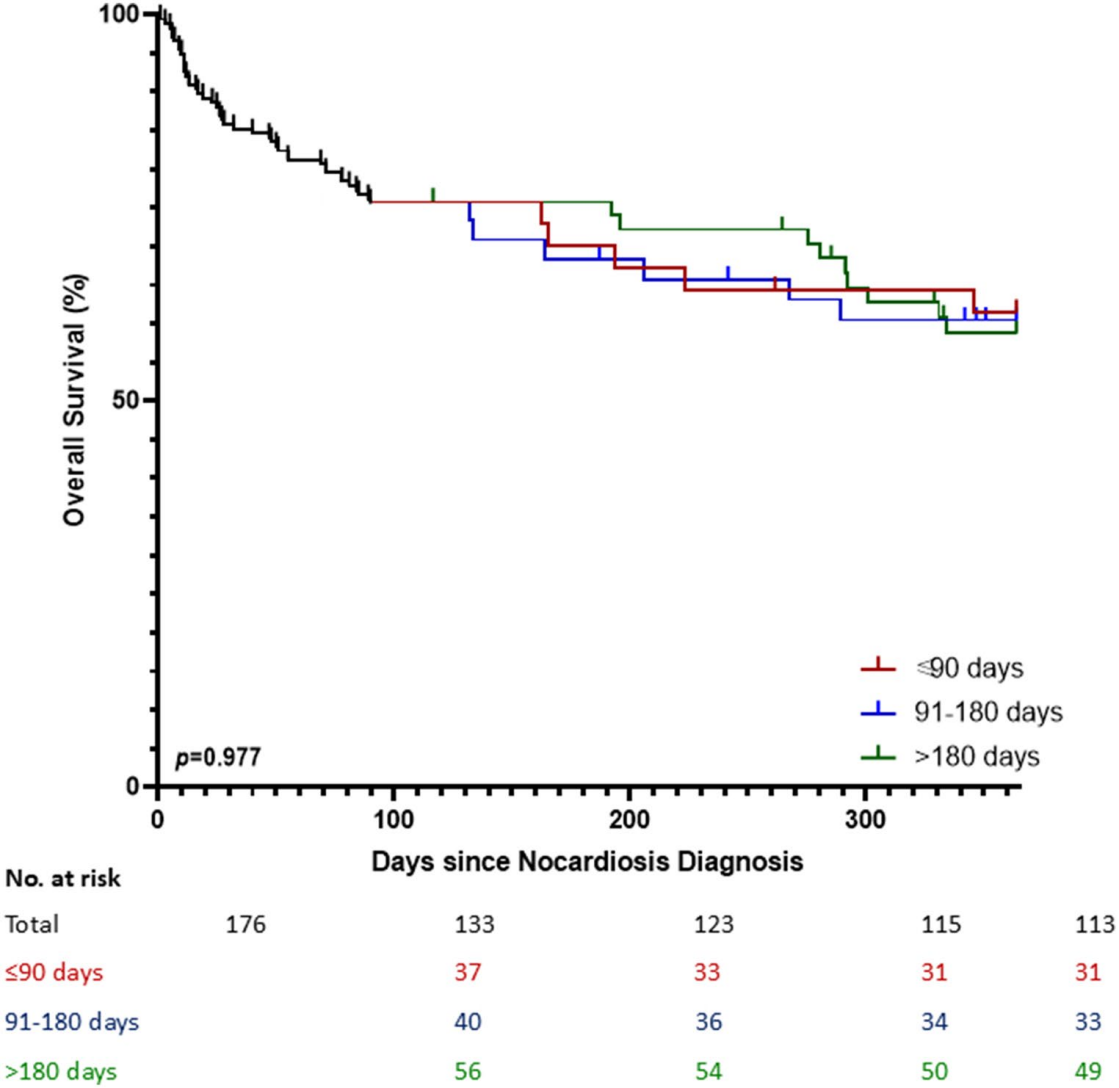
Survival curves of the cohort, according to the treatment group. The entire cohort is presented as a single curve up to 90 days, then divided into 3 treatment groups, according to the total treatment duration. *p* value calculated using Log-rank test

One-year all-cause mortality was documented for 20/133 (15%) individuals who survived at least 90 days following nocardiosis diagnosis. Mortality was not associated with treatment duration groups (*p* = 0.977, Fig. 3), and mortality rates were similar in the three groups (14%, 15%, and 16%, for short, intermediate, and prolonged treatment duration, respectively, *p* = 0.945) (Table 1).

Details of the 20 individuals who died within one year are shown in Table 2. Ten (50%) of them died while still receiving antibiotic therapy for nocardiosis. Data on death circumstances were available for 17/20 (85%), while the cause of death could be determined for 14/20 (70%). Of these, only one (7%) individual died from nocardiosis.

**Table 2:** Characteristics and causes of death of the 20 individuals who died between 3–12 months following nocardiosis diagnosis

Serial number	Gender	Age	Comorbidities and immunomodulatory agents	Immune suppression	Nocardiosis syndrome	Nocardiosis treatment duration (days)	Died while still receiving treatment	Diagnosis-to-death interval (months)	Immediate cause of death
1	Man	78	AIHA, MDS, COPD; prednisone (70 mg per day)	Substantial	Disseminated without CNS involvement	126	Yes	4	Unavailable data
2	Woman	73	CLL; fludarabine, cyclophosphamide, prednisone (40 mg per day)	Substantial	Solitary pulmonary	140	Yes	5	Unavailable data
3	Man	61	MM, autologous bone marrow transplant recipient	Substantial	Disseminated with CNS involvement	189	Yes	6	MM progression
4	Woman	38	Bilateral lung transplant recipient due to IPF, chronic mechanical ventilation, polymyositis; cyclosporine, mycophenolate mofetil, prednisone (40 mg per day)	Substantial	Solitary pulmonary	133	Yes	4	Severe pneumonia
5	Man	68	Single lung transplant recipient due to IPF; tacrolimus, mycophenolate mofetil, prednisone (20 mg per day)	Substantial	Solitary pulmonary	266	Yes	9	Lung transplant rejection
6	Woman	68	Kidney transplant recipient with chronic rejection, type II DM, ESRD with hemodialysis; tacrolimus, mycophenolate mofetil, prednisone (5 mg per day)	Substantial	Solitary pulmonary	287	Yes	9	Undetermined
7	Man	35	Bilateral lung transplant recipient due to CF; tacrolimus, mycophenolate mofetil, prednisone (30 mg per day)	Substantial	Disseminated with CNS involvement	196	Yes	6	Nocardiosis
8	Man	52	Bilateral lung transplant recipient due to progressive systemic sclerosis, ESRD with hemodialysis, CHF; tacrolimus, prednisone (10 mg per day)	Substantial		259	No	10	Decompensated heart failure
9	Woman	72	Mycosis fungoides with large cell transformation; pralatrexate, prednisone (15 mg per day)	Substantial	Solitary cutaneous	84	No	7	Severe pneumonia
10	Man	54	Primary myelofibrosis, allogeneic bone marrow transplant recipient with chronic GVHD; cyclosporine, ruxolitinib, mycophenolate mofetil, prednisone (30 mg per day)	Substantial	Disseminated with CNS involvement	224	Yes	9	Undetermined
11	Woman	66	Ulcerative colitis, s/p tricuspid and mitral valves repair repair; infliximab, prednisone (30 mg per day)	Substantial	Disseminated with CNS involvement	77	No	5	Urosepsis
12	Man	53	Metastatic small cell lung cancer; carboplatin and etoposide	Mild-moderate	Solitary pulmonary	168	No	9	Lung cancer progression
13	Man	56	AIDS (24 CD4 + cells per µL) with opportunistic infections (*Pneumocystis jirovecii* pneumonia, toxoplasmosis, and Cryptococcal meningitis)	Substantial	Solitary pulmonary	35	No	5	Cryptococcosis with CNS involvement
14	Man	81	MM with leukemic transformation; ixazomib, lenalidomide, dexamethasone (20 mg per day)	Substantial	Disseminated with CNS involvement	287	Yes	9	Plasma cell leukemia
15	Man	65	COPD	Nonapparent	Disseminated with CNS involvement	7^a^	No	12	Severe pneumonia caused by ESBL-producing *K. pneumoniae*
16	Man	70	DLBCL, allogenous bone marrow transplant recipient; ibrutinib, prednisone (10 mg per day)	Substantial	Solitary pulmonary	336	Yes	11	Lymphoma progression
17	Man	69	MDS, marginal zone lymphoma; ibrutinib, prednisone (10 mg per day)	Substantial	Solitary pulmonary	105	No	6	Lymphoma progression
18	Woman	77	Chronic mechanical ventilation due to subarachnoid hemorrhage	None	Solitary pulmonary	77	No	6	Unavailable data
19	Woman	67	SLE with pulmonary fibrosis; prednisone (10 mg per day)	Mild-moderate	Solitary pulmonary	196	No	10	Undetermined
20	Woman	64	Metastatic ovarian adenocarcinoma, bronchiectasis; carboplatin	Mild-moderate	Solitary pulmonary	175	No	10	*K. pneumoniae* bacteremia during neutropenia

^a^ The patient was diagnosed with a single brain abscess caused by *Nocardia farcinica*, without lung or skin involvement. Following successful abscess drainage, the patient was treated intravenously with two agents. At time of discharge, the patient refused to continue with additional antibiotic therapy. Upon readmission due to severe ESBL-producing *K. pneumoniae* pneumonia, brain CT confirmed that the abscess has resolved

AIDS = acquired immunodeficiency syndrome; AIHA = Autoimmune hemolytic anemia; CF = cystic fibrosis; CHF = congestive heart failure; CLL = Chronic lymphocytic leukemia; CNS = central nervous system; COPD = chronic obstructive pulmonary disease; DLBCL = diffuse large B-cell lymphoma; ESRD = end stage renal disease; DM = diabetes mellitus; IPF = idiopathic pulmonary fibrosis; MDS = myelodysplastic syndrome; MM = multiple myeloma; SAH = subarachnoid hemorrhage; SLE = systemic lupus erythematosus

One-year nocardiosis relapse occurred among 2/133 (1.5%) individuals (Table 1), both treated for long duration with suboptimal regiments (for further details, see supplementary Table S5).

A total of 49/133 (37%) of the cohort had at least one antibiotic-related side effect. Sixteen (12%) and 19 (14%) were diagnosed with renal and bone marrow toxicity, respectively; 6 (5%) were diagnosed with *Clostridioides difficile* infection; and 12 (9%) individuals developed allergic reactions. The proportions of the side effects did not differ between the treatment duration groups (Table 1).

### Sensitivity analyses

Reanalyzing the cohort following an exclusion of 49 individuals who died while on treatment (43 and 6 of whom died within three months and between 3 and 12 months from diagnosis, respectively) yielded similar results (Supplementary Table S6). Reclassifying the treatment duration groups using different cutoffs, with short duration defined as 120 days and intermediate duration as 121–180 days, yielded similar results, with the addition of statistically significant associations between treatment duration and hematopoietic stem cell transplantation (*p* = 0.016) and Nocardia species (*p =* 0.031) (supplementary Table S7). Using a single grouping cutoff at 6 months, the results remained similar, while treatment duration did correlate with Nocardia species (*p =* 0.038) (supplementary Table S8).

### Solitary pulmonary nocardiosis

A total of 73/133 (55%) individuals had solitary pulmonary nocardiosis; 20 (27%) of them were apparently immunocompetent, receiving short (*N* = 8, 40%), intermediate (*N* = 6, 30%), and prolonged (*N* = 6, 30%) treatment durations. None have died within one year from diagnosis.

Among the 53 individuals with immune suppression, 1-year mortality rates were 31% (5/16) and 16% (6/37) for mild-moderate and substantial immune suppression, respectively. Treatment duration did not correlate with mortality for either mild-moderate (*p* = 0.808) or substantial (*p* = 0.999) immune suppression (Table 3).

**Table 3:** One-year all-cause mortality of individuals with solitary pulmonary nocardiosis, according to their immune status and treatment duration

Treatment duration (days)	Total *N* = 73 (100%)	Status at 1-year	Apparently immunocompetent *N* = 20 (27%)	Mild-moderate immune suppression ^a^ *N* = 16 (22%)	Substantial immune suppression ^b^ *N* = 37 (51%)
**≤ 90**	*N* = 20 (27%)	**Survived**	8	4	6
		**Died**	0	1 (20.0)	1 (14.3)
**91–180**	*N* = 25 (34%)	**Survived**	6	4	10
		**Died**	0	3 (42.9)	2 (16.7)
**> 180**	*N* = 28 (38%)	**Survived**	6	3	15
		**Died**	0	1 (25.0)	3 (16.7)
**One-year overall mortality**			0	5 (31.2)	6 (16.2)

^a^ Individuals with active malignancy, primary immune deficiency, autoimmune diseases, and chronic corticosteroid therapy (≥ 5 and < 20 mg per day); ^b^ Solid organ transplant recipients, hematopoietic stem cell transplant recipients, and individuals with acquired immunodeficiency syndrome (AIDS) or on chronic corticosteroid therapy (≥ 20 mg per day)

### Solitary lymphocutaneous nocardiosis

Twenty-three individuals were diagnosed with solitary lymphocutaneous nocardiosis. They tended to have lesser immune suppression: 15 (65%) were apparently immunocompetent, while 6 (26%) and 2 (9%) had mild to moderate and substantial immune suppression, respectively. Most of these patients were treated for shorter periods: 11 (48%) of them for ≤ 90 days; 3 (13%) for 91–120 days; 6 (26%) for 121–180 days and only 3 (13%) received prolonged treatment duration (> 180 days). By 1-year, 1/23 (4%) of these patients died (at 224 days from diagnosis, due to leukemic transformation of mycosis fungoides). There were no cases of nocardiosis relapse.

## Discussion

In this retrospective multi-center study assessing 176 individuals with nocardiosis, short (≤ 90 days) treatment duration was given to approximately a quarter of the cohort and was not associated with a higher relapse or increased mortality rates at one year following diagnosis. These individuals tended to have less immune suppression, a more localized infection, and lower proportions of CNS involvement or *N. farcinica* as the causative species.

These findings reflect that clinicians have shortened antibiotic courses in selected cases based on the clinical scenario and patient characteristics. Deaths related to nocardiosis predominantly occurred at an early stage, primarily during the first 3 months, while on therapy. The relatively low probability for death related to nocardiosis beyond the initial phase may support the decision to shorten treatment duration for patients with an adequate initial response to therapy.

Risk factors for poorer outcomes in nocardiosis vary across patient populations. While among allogeneic HCTs with nocardiosis, failure to achieve complete remission of the underlying diseases and concurrent or prior bacterial infection were identified as risk factors for mortality; [7] among SOTRs, malignancy, recent fungal infection, donor’s age, and acute rejection all independently conferred a greater risk for mortality [6]. 

Beyond the interplay between host-pathogen factors, nocardiosis clinical syndrome also plays a prognostic role, as dissemination is strongly associated with mortality [10, 11]. As for the causative species, *N. farcinica* is associated with CNS involvement and consequent poorer prognosis in comparison to other species [12]. 

The heterogeneity in treatment durations observed in our study emphasizes that although recommendations advocate a prolonged antibiotic course for nocardiosis, the clinicians assess the individual risk on a case-by-case basis. The effectiveness of the short treatment courses provided to a non-negligible portion of the cohort suggests that tailor-made treatment duration is widely practiced in real life, likely by combining clinical assessment with existing evidence on factors for poorer outcomes. Considering that an RCT is unfeasible, our findings encourage this clinical approach.

The use of 18-fluorodeoxyglucose Positron Emission Tomography/Computed Tomography (FDG-PET/CT) has been recently demonstrated to contribute to the management of patients with nocardiosis, including affecting decisions on oral switch and duration of therapy [13]. Use of FDG-PET/CT as part of management algorithms tailoring treatment to individual patients, has been also shown for other infections [14]. Similarly, biomarkers, such as C-related protein (CRP) and procalcitonin, have been demonstrated to be safely used for shortening treatment duration in bacterial infections [15]. Strategies to personalize the duration of therapy in nocardiosis using these tests as part of the evaluation should be investigated.

The small case series that have laid the foundations for recommending prolonged treatment duration for nocardiosis were published in approximately 50 years ago and assessed outcomes of SXT, or merely sulfamethoxazole or another sulfonamide, as monotherapy [3, 4]. Throughout the years, combination treatment gained dominance, recognizing the synergism between sulfonamides and beta-lactams against Nocardia species [16]. In our study, most (> 80%) patients were treated with SXT based combination regimens. Since these case series were published, new laboratory methods have enabled rapid identification of Nocardia at the species level, with more advanced and rapid antimicrobial susceptibility testing (AST) for direct therapy. As most frequent pathogenic species (i.e., *N. cyriacigeorgica* and *N. farcinica*) demonstrate typical resistance patterns for preferred beta-lactam agents [2], rapid species identification has facilitated the choice of an accompanying drug empirically (i.e., before AST). Accordingly, it is likely that rapid species identification and efficient AST, together with combination regimen during the initial phase, increase treatment effectiveness and facilitate a shorter treatment duration.

The sub-group of individuals with solitary pulmonary nocardiosis is of particular interest. In our study, 27% (20/73) were apparently immunocompetent, in line with previous reports demonstrating similar proportions of immunocompetent individuals, frequently with an underlying pulmonary comorbidity [17, 18]. We found no cases of death or relapse, regardless of treatment duration. This implies that for the non-negligible sub-population of immunocompetent individuals with pulmonary nocardiosis, a treatment duration of six months is likely unnecessary. Moreover, of the 12 individuals with immune suppression and pulmonary nocardiosis receiving short treatment duration (≤ 3 months), there were only 2 (17%) cases of death, both unrelated to nocardiosis. This reinforces the concept that although treatment guidance documents tend to recommend treatment regimens of at least six months for this population, 3–6 months should also be safe for these individuals. Further studies should focus on and describe the clinical anchors facilitating their identification (e.g., good response to treatment, rapidly decreasing markers of inflammation, resolved lesions on imaging, etc.).

Shortening antibiotic treatment has been demonstrated to diminish antimicrobial resistance emergence and decrease the burden of antibiotic side effects and costs [19]. No difference in adverse events was demonstrated in our study between treatment duration groups. This could be a realistic finding or merely reflect diminished statistical power for the secondary outcomes.

Our study has some limitations; although this is a multi-center study, all data was obtained from only one country; lack of data on biomarkers measurements and imaging studies throughout the course of treatment may have contributed to duration planning; and lack of power to address adverse events. Given the heterogeneous nature of Nocardia infections, the limited sample size, and the retrospective design, we cannot definitively conclude non inferiority of short treatment duration for specific patients’ populations. Nevertheless, our findings suggest that for some populations, shorter therapy is feasible and should be considered along with close follow-up (primarily for solitary lymphocutaneous and solitary pulmonary nocardiosis).

In conclusion, short antibiotic therapy may be appropriate for some patients with nocardiosis. These include patients with less intensive immune suppression, those with a localized Nocardia infection without CNS involvement, and those with Nocardia species other than *N. farcinica*. Further studies should investigate strategies to individualize the duration of therapy for the heterogeneous population of nocardiosis patients.

## Electronic supplementary material

Below is the link to the electronic supplementary material.

Supplementary Material 1

## Data Availability

The datasets used and analyzed during the current study are available from the corresponding author on reasonable request.

## Declarations

### Generative AI in scientific writing

We did not use artificial intelligence tools during the preparation of this work.
