# Supplementary material for: Shorter vs. standard-duration antibiotic therapy for nocardiosis: a multi-center retrospective cohort study
Source: Infection. 2024 Nov 26;53(3):1115–27. doi: 10.1007/s15010-024-02445-0 (PMC12137364; doi:10.1007/s15010-024-02445-0)
Supplement: Supplementary file 1 — Supplementary Material 1 [file 15010_2024_2445_MOESM1_ESM.docx]

**Shorter *vs* standard-duration antibiotic therapy for nocardiosis: a multicenter retrospective cohort study**

*** Supplementary material ***

**Contents**

[**Table S1. General characteristics of participating medical centers** 2](#_Toc179918474)

[**Table S2. Definitions for each of the antibiotic-related adverse event types** 2](#_Toc179918475)

[**Table S3. Microbiological methods implemented for *Nocardia* isolation, identification and antimicrobial susceptibility testing** 2](#_Toc179918476)

[**Table S4. Characteristics and causes of death of the 43 individuals who died within 90 days from nocardiosis diagnosis** 3](#_Toc179918477)

[**Table S5. Characteristics and treatment regimens of the two individuals who relapsed by 1-year** 4](#_Toc179918478)

[**Table S6. Clinical characteristics and outcomes of individuals diagnosed with nocardiosis, according to treatment duration range (sensitivity analysis by exclusion of individuals who died while on anti-Nocardial treatment [N=49])** 5](#_Toc179918479)

[**Table S7. Clinical characteristics and outcomes of individuals diagnosed with nocardiosis, according to treatment duration range: sensitivity analysis using a minimal cutoff of 120 days** 6](#_Toc179918480)

[**Table S8. Clinical characteristics and outcomes of individuals diagnosed with nocardiosis, according to treatment duration range: sensitivity analysis using a cutoff of 180 days** 7](#_Toc179918481)

# **Table S1. General characteristics of participating medical centers**

| **Medical Center** | **Rabin Medical Center, Beilinson Hospital** | **Sheba Medical Center** | **Rambam Healthcare Campus** | **Soroka Medical Center** |
| --- | --- | --- | --- | --- |
| Period of patients’ identification (for which data on *Nocardia* isolations could be retrieved) | 2007-2022 | 2012-2022 | 2010-2022 | 2012-2022 |
| City | Petah Tikva | Ramat Gan | Haifa | Be’ersheba |
| Tertiary hospital | Yes | Yes | Yes | Yes |
| Number of acute-care hospital beds | 835 | 1116 | 930 | 1123 |
| University affiliation | Tel Aviv University | Tel Aviv University | Technion-Israel Institute of Technology | Ben-Gurion University of the Negev |

# **Table S2. Definitions for each of the antibiotic-related adverse event types**

| **Type of antibiotic-related adverse event** | **Definition** |
| --- | --- |
| *Clostridioides* *difficile* infection | Acute diarrhea microbiologically confirmed by positive *Clostridioides* *difficile* stool test (enzyme-linked immunoassay for two bacterial antigens [glutamate dehydrogenase and toxin B] and/or by polymerase chain reaction), that has led to treatment (oral vancomycin, fidaxomicin, or metronidazole). |
| Renal toxicity | Acute kidney injury, defined according to the AKIN criteria as increase in serum creatinine of ≥0.3 mg/dL or ≥50% within 48 hours compared to baseline, OR Urine output of <0.5 mL/kg/hour for >6 hours. |
| Bone marrow toxicity | Decline in either white blood cell count, absolute neutrophil count, absolute lymphocyte count, serum hemoglobin, or thrombocytes, unrelated to another mechanism (*e.g*., acute bleeding or anti-cancer therapy), leading to treatment cessation and/or switch to another antibiotic agent. |
| Allergic reactions | Immediate reactions, occurring within one hour following first administered dose OR delayed reactions, occurring after one hour (usually more than six hours and occasionally weeks to months) following first administration. Any drug related cutaneous, mucocutaneous, or respiratory phenomenon that has led to treatment cessation and/or switch to another antibiotic agent. |

# **Table S3. Microbiological methods implemented for *Nocardia* isolation, identification and antimicrobial susceptibility testing**

| **Medical Center** | **Rabin Medical Center, Beilinson Hospital** | **Sheba Medical Center** | **Rambam Healthcare Campus** | **Soroka Medical Center** |
| --- | --- | --- | --- | --- |
| **MALDI TOF MS is implemented for routine *Nocardia* identification since** | 2015 | 2021 | 2015 | 2013 |
| **Sequencing for *Nocardia* species identification** | In selected cases | Yes | Yes | No |
| Since when (year)? | 2020 | 2022 | 2003 |  |
| Gene used for sequencing | Hsp65 | 16S | Hsp65 |  |
| **Routine AST for *Nocardia*?** | Yes | Yes | Yes | No |
| Performed at the institutional laboratory? | No | Yes | Until 2018 |  |
| If performed at another center, where? | Tel Aviv Sourasky Medical Center | No | Tel Aviv Sourasky Medical Center |  |
| AST methods performed (BMD, E-test) | BMD (*Sensititre*™ *NOCARDIA*) | E-test – until XX; thereafter: BMD (*Sensititre*™ *NOCARDIA*) | Until 2018: E-test.  Since 2018: (*Sensititre*™ *NOCARDIA*) |  |

AST = antimicrobial susceptibility testing; BMD = broth microdilution; MALDI TOF MS = matrix-assisted laser desorption/ionization time-of-flight mass spectrometer.

# **Table S4. Characteristics and causes of death of the 43 individuals who died within 90 days from nocardiosis diagnosis**

| **Serial number** | **Gender** | **Age** | **Main underlying condition** | **Immune suppression** | **Nocardiosis syndrome** | **Diagnosis-to-death interval (days)** | **Immediate cause of death** |
| --- | --- | --- | --- | --- | --- | --- | --- |
| 1 | Male | 30 | Kidney transplant recipient | Substantial | Disseminated with CNS involvement | 50 | Unknown |
| 2 | Male | 71 | Chronic obstructive pulmonary disease | Mild-moderate | Solitary pulmonary | 25 | Unknown |
| 3 | Female | 53 | Interstitial lung disease | Substantial | Disseminated with CNS involvement | 12 | Unknown |
| 4 | Female | 76 | Sudden cardiac death and mechanical ventilation | Nonapparent | Solitary pulmonary | 71 | Unknown |
| 5 | Male | 71 | Metastatic non-small cell lung cancer | Substantial | Solitary pulmonary | 7 | Unknown |
| 6 | Male | 56 | Allogeneic hematopoietic stem cell transplant recipient with relapsed acute myeloid leukemia | Substantial | Disseminated with CNS involvement | 78 | Pulmonary aspergillosis |
| 7 | Female | 67 | Chronic obstructive pulmonary disease | Substantial | Solitary pulmonary | 1 | Severe pneumonia |
| 8 | Female | 65 | Kidney transplant recipient | Substantial | Solitary pulmonary | 48 | Candidemia |
| 9 | Male | 26 | Heart and lung transplant recipient | Substantial | Solitary pulmonary | 90 | Carbapenem-resistant *A. baumannii* pneumonia |
| 10 | Male | 59 | Lung transplant recipient | Substantial | Solitary pulmonary | 55 | Severe pneumonia |
| 11 | Male | 78 | Metastatic non-small cell lung cancer | Substantial | Solitary pulmonary | 32 | Malignancy |
| 12 | Female | 75 | Chronic lymphocytic leukemia | Substantial | Disseminated with CNS involvement | 69 | Unknown |
| 13 | Male | 77 | Metastatic non-small cell lung cancer | Substantial | Solitary pulmonary | 11 | **Nocardiosis** |
| 14 | Male | 86 | Sick sinus syndrome with pacemaker insertion | Nonapparent | Disseminated without CNS involvement | 23 | **Nocardiosis** |
| 15 | Male | 80 | Marginal cell lymphoma | Substantial | Disseminated without CNS involvement | 11 | **Nocardiosis** |
| 16 | Female | 74 | Chronic obstructive pulmonary disease | Substantial | Solitary pulmonary | 3 | **Nocardiosis** |
| 17 | Male | 73 | Chronic lymphocytic leukemia and bronchiectasis | Mild-moderate | Solitary pulmonary | 13 | Malignancy |
| 18 | female | 85 | Chronic dermatitis | Substantial | Disseminated with CNS involvement | 27 | **Nocardiosis** |
| 19 | male | 30 | Allogeneic hematopoietic stem cell transplant recipient with chronic graft versus host disease | Substantial | Solitary pulmonary | 81 | Severe nosocomial pneumonia |
| 20 | Female | 66 | Metastatic non-small cell lung cancer | Substantial | Solitary pulmonary | 47 | Malignancy |
| 21 | Male | 87 | Metastatic squamous cell carcinoma | Substantial | Solitary pulmonary | 40 | COVID-19 |
| 22 | Male | 52 | Acquired immunodeficiency syndrome | Nonapparent | Disseminated with CNS involvement | 17 | Intracranial hemorrhage |
| 23 | Male | 66 | Acute myeloid leukemia | Substantial | Solitary pulmonary | 11 | Malignancy |
| 24 | Male | 85 | Metastatic adenocarcinoma of colon | Substantial | Solitary pulmonary | 10 | Methicillin-sensitive *S. aureus* bacteremic pneumonia |
| 25 | female | 89.9 | Atrial fibrillation and cerebrovascular disease | Nonapparent | Solitary pulmonary | 9 | **Nocardiosis** |
| 26 | Male | 50.6 | Allogeneic hematopoietic stem cell transplant recipient with chronic graft versus host disease | Substantial | Osteomyelitis | 89 | **Nocardiosis** |
| 27 | male | 67 | Chronic obstructive pulmonary disease | Substantial | Disseminated with CNS involvement | 19 | **Nocardiosis** |
| 28 | female | 64 | Nephrotic syndrome | Substantial | Disseminated with CNS involvement | 16 | **Nocardiosis** |
| 29 | female | 77 | Rheumatoid arthritis | Mild-moderate | Disseminated with CNS involvement | 6 | **Nocardiosis** |
| 30 | male | 44 | Bronchiectasis | Nonapparent | Solitary pulmonary | 26 | **Nocardiosis** |
| 31 | male | 58 | T-cell lymphoma | Mild-moderate | Disseminated with CNS involvement | 84 | Malignancy |
| 32 | Male | 81 | Multiple myeloma | Mild-moderate | Disseminated with CNS involvement | 78 | Malignancy |
| 33 | Male | 84 | Cirrhosis (non-alcoholic) | Substantial | Disseminated with CNS involvement | 5 | **Nocardiosis** |
| 34 | Male | 75 | Metastatic non-small cell lung cancer | Substantial | Solitary pulmonary | 13 | *E. coli* bacteremia |
| 35 | Male | 64 | Metastatic transitional cell cancer | Substantial | Disseminated without CNS involvement | 51 | Malignancy |
| 36 | Male | 76 | Chronic lymphocytic leukemia | Mild-moderate | Disseminated with CNS involvement | 71 | **Nocardiosis** |
| 37 | Female | 76 | Metastatic small cell lung cancer | Mild-moderate | Solitary pulmonary | 55 | Malignancy |
| 38 | Male | 44 | Metastatic non-small cell lung cancer | Substantial | Disseminated with CNS involvement | 26 | Malignancy |
| 39 | Male | 47 | Allogeneic hematopoietic stem cell transplant recipient | Substantial | Solitary pulmonary | 28 | **Nocardiosis** |
| 40 | Male | 35 | Metastatic sarcoma | Mild-moderate | Solitary pulmonary | 11 | Malignancy |
| 41 | Female | 58 | Diffuse large B-cell lymphoma | Substantial | Disseminated with CNS involvement | 85 | Difficult to treat *P.* *aeruginosa* bacteremia |
| 42 | Female | 65 | Metastatic adenocarcinoma of breast | Substantial | Solitary pulmonary | 6 | **Nocardiosis** |
| 43 | Male | 67 | Metastatic non-small cell lung cancer | Substantial | Solitary pulmonary | 9 | **Nocardiosis** |

# **Table S5. Characteristics and treatment regimens of the two individuals who relapsed by 1-year**

| **Serial number** | **Gender** | **Age** | **Comorbidities and immunomodulatory agents** | **Immune suppression** | **Nocardiosis syndrome** | **Treatment regimen** | **Diagnosis-to-relapse interval (months)** |
| --- | --- | --- | --- | --- | --- | --- | --- |
| 1 | Man | 60 | Chronic lymphocytic leukemia with hypogammaglobinemia; ibrutinib, intravenous immunoglobulin G transfusions | Substantial | Disseminated without CNS involvement | SXT monotherapy | 6 |
| 2 | Woman | 36 | Malignant thymoma, paraneoplastic myasthenia gravis; Prednisone (5 mg per day) | Mild-moderate | Disseminated with CNS involvement | Minocycline and clarithromycin | 9 |

# **Table S6. Clinical characteristics and outcomes of individuals diagnosed with nocardiosis, according to treatment duration range (sensitivity analysis by exclusion of individuals who died while on anti-Nocardial treatment [N=49])**

|  | **Died while on therapy**  **N = 49 (28%)** | **Treatment duration** | | | ***p* ^a^** |
| --- | --- | --- | --- | --- | --- |
|  |  | **≤90 days**  **N = 37 (29%)** | **91-180 days**  **N = 37 (%)** | **>180 days**  **N = 53 (42%)** |  |
| **Demographics and comorbidities** |  |  |  |  |  |
| Age at diagnosis, median (IQR) | 67 (20) | 56 (30) | 66 (25) | 60 (24) | 0.390 |
| Female gender, N (%) | 17 (34.7) | 17 (45.9) | 15 (40.5) | 25 (47.2) | 0.814 |
| Charlson score, median (IQR) | 4.0 (4.0) | 2.0 (3.0) | 2.0 (3.0) | 2.0 (4.0) | 0.413 |
| Solid organ transplant recipient, N (%) | 6 (12.2) | 2 (5.4) | 7 (18.9) | 9 (17.0) | 0.186 |
| Hematopoietic stem cell transplant recipient, N (%) | 7 (14.3) | 1 (2.7) | 4 (10.8) | 7 (13.2) | 0.239^⸙^ |
| Chronic pulmonary disease, N (%) | 16 (32.7) | 13 (35.1) | 16 (43.2) | 25 (47.2) | 0.521 |
| Malignancy, N (%) | 29 (59.2) | 8 (21.6) | 9 (24.3) | 15 (28.3) | 0.765 |
| Autoimmune disease, N (%) | 9 (18.4) | 5 (13.5) | 6 (16.2) | 14 (26.4) | 0.260 |
| Primary immune deficiency, N (%) | 0 | 2 (5.4) | 2 (5.4) | 0 | 0.219^⸙^ |
| Diabetes mellitus, N (%) | 20 (40.8) | 11 (29.7) | 12 (32.4) | 19 (35.8) | 0.828 |
| Systemic corticosteroid therapy, N (%) | 37 (75.5) | 11 (29.7) | 20 (54.1) | 32 (60.4) | **0.014** |
| Prednisone equivalent dose, median (IQR) ^b^ | 27 (32) | 30 (35) | 18 (32) | 30 (44) | 0.087 |
| Prednisone >20 mg per days, N (%) | 31 (63.3) | 7 (18.9) | 10 (27.0) | 24 (45.3) | **0.023** |
| Immune status |  |  |  |  | **0.026** |
| Apparently immunocompetent, N (%) | 5 (10.2) | 19 (51.4) | 12 (32.4) | 14 (26.4) |  |
| Mild-moderate immune suppression ^c^, N (%) | 8 (16.3) | 9 (24.3) | 9 (24.3) | 8 (15.1) |  |
| Substantial immune suppression ^d^, N (%) | 36 (73.5) | 9 (24.3) | 16 (43.2) | 31 (58.5) |  |
| **Nocardiosis** |  |  |  |  |  |
| *Nocardia* species ^e^ |  |  |  |  | 0.067^⸙^ |
| *Nocardia cyriacigeorgica*, N (%) | 14 (51.9) | 10 (50.0) | 11 (57.9) | 12 (41.4) |  |
| *Nocardia farcinica*, N (%) | 7 (25.9) | 1 (5.0) | 3 (15.8) | 8 (27.6) |  |
| *Nocardia otitidiscaviarum*, N (%) | 3 (11.1) | 0 | 1 (5.3) | 3 (10.3) |  |
| *Nocardia brasiliensis*, N (%) | 0 | 5 (25.0) | 1 (5.3) | 0 |  |
| Miscellaneous *Nocardia* spp., N (%) | 3 (11.1) | 4 (20.0) | 3 (15.8) | 6 (20.7) |  |
| Nocardiosis syndrome |  |  |  |  | **<0.001^⸙^** |
| Solitary lymphocutaneous, N (%) | 0 | 11 (29.7) | 9 (24.3) | 3 (5.7) |  |
| Osteomyelitis, N (%) | 1 (2.0) | 1 (2.7) | 1 (2.7) | 0 |  |
| Solitary pulmonary, N (%) | 28 (57.1) | 20 (54.1) | 23 (62.2) | 26 (49.1) |  |
| Disseminated, no CNS involvement, N (%) | 4 (8.2) | 3 (8.1) | 1 (2.7) | 10 (18.9) |  |
| Disseminated, CNS involvement, N (%) | 16 (32.7) | 2 (5.4) | 3 (8.1) | 14 (26.4) |  |
| Oxygen supplementation during hospitalization |  |  |  |  | 0.558^⸙^ |
| No need, N (%) | 12 (26.1) | 23 (74.2) | 25 (75.8) | 33 (63.5) |  |
| Nasal canula, N (%) | 11 (23.9) | 3 (9.7) | 6 (18.2) | 10 (19.2) |  |
| High-flow nasal canula, N (%) | 4 (8.7) | 1 (3.2) | 0 | 4 (7.7) |  |
| Non-invasive ventilation, N (%) | 2 (4.3) | 0 | 0 | 0 |  |
| Mechanical ventilation, N (%) | 17 (37.0) | 4 (12.9) | 2 (6.1) | 5 (9.6) |  |
| C-reactive protein at presentation (mg/dL), median (IQR) | 12.3 (17.0) | 1.9 (17.0) | 8.7 (13.0) | 9.1 (18.0) | 0.269 |
| **Treatment** |  |  |  |  |  |
| SXT-based regimen, N (%) | 41 (83.7) | 29 (78.4) | 33 (89.2) | 45 (84.9) | 0.436 |
| Combination treatment, N (%) | 38 (77.6) | 16 (43.2) | 26 (70.3) | 43 (81.1) | **<0.001** |
| Combination treatment duration (weeks), median (IQR) | 2.0 (6.1) | 6.3 (7.8) | 6.0 (9.1) | 14.5 (27.0) | **<0.001** |
| IV therapy duration (weeks), median (IQR) | 2.0 (7.3) | 0.8 (5.5) | 3.0 (11.0) | 5.0 (23.5) | **<0.001** |
| Antibiotic-related side effects, N (%) | 21 (42.9) | 12 (32.4) | 15 (40.5) | 17 (32.1) | 0.669 |
| Renal toxicity, N (%) | 7 (16.3) | 2 (5.7) | 6 (17.6) | 7 (13.2) | 0.302^⸙^ |
| Bone marrow toxicity, N (%) | 10 (23.3) | 5 (14.3) | 7 (19.4) | 6 (11.5) | 0.586 |
| *C. difficile* infection, N (%) | 2 (4.7) | 2 (5.7) | 1 (2.9) | 2 (3.8) | 0.999^⸙^ |
| Allergic reaction, N (%) | 0 | 3 (8.6) | 4 (11.4) | 5 (9.6) | 0.999^⸙^ |
| Surgical intervention, N (%) | 5 (11.1) | 8 (22.2) | 8 (22.2) | 13 (25.0) | 0.937 |
| **Outcomes at one year from diagnosis** |  |  |  |  |  |
| Rehospitalization, N (%) | NA | 17 (45.9) | 17 (45.9) | 27 (50.9) | 0.857 |
| Nocardiosis relapse, N (%) | NA | 0 | 0 | 2 (3.8) | 0.510^⸙^ |
| All-cause mortality, N (%) | NA | 5 (13.5) | 3 (8.1) | 6 (11.3) | 0.727^⸙^ |

^a^ Calculated for the three treatment groups using Chi-square test or Fisher’s exact test (^⸙^) and one-way ANOVA for categorical and continuous variables, respectively; ^b^ Average daily prednisone dosage (in milligrams), during the 90 days prior to presentation. Whenever dexamethasone was used, dosage was converted to the equivalent prednisone dosage; ^c^ Individuals with active malignancy, primary immune deficiency, autoimmune diseases, and chronic corticosteroid therapy (≥5 and <20 mg per day); ^d^ Solid organ transplant recipients, hematopoietic stem cell transplant recipients, and individuals with acquired immunodeficiency syndrome (AIDS) or on chronic corticosteroid therapy (≥20 mg per day); ^e^ Identification to the species level was available for 95/176 (54%) of the cohort. IV = intravenous; NA = not applicable; SXT = sulfamethoxazole-trimethoprim.

# **Table S7. Clinical characteristics and outcomes of individuals diagnosed with nocardiosis, according to treatment duration range: sensitivity analysis using a minimal cutoff of 120 days**

|  | **Died within**  **90 days**  **N = 43 (24%)** | **Treatment duration** | | | ***p* ^a^** |
| --- | --- | --- | --- | --- | --- |
|  |  | **≤120 days**  **N = 53 (40%)** | **121-180 days**  **N = 24 (18%)** | **>180 days**  **N = 56 (42%)** |  |
| **Demographics and comorbidities** |  |  |  |  |  |
| Age at diagnosis, median (IQR) | 67 (21) | 61 (23) | 58 (29) | 61 (22) | 0.990 |
| Female gender, N (%) | 14 (32.6) | 25 (47.2) | 9 (37.5) | 26 (46.4) | 0.708 |
| Charlson score, median (IQR) | 4 (4) | 2.0 (2.0) | 2.5 (3.0) | 2.0 (3.0) | 0.255 |
| Solid organ transplant recipient, N (%) | 4 (9.3) | 6 (11.3) | 4 (16.7) | 10 (17.9) | 0.564^⸙^ |
| Hematopoietic stem cell transplant recipient, N (%) | 5 (11.6) | 1 (1.9) | 4 (16.7) | 9 (16.1) | **0.016^⸙^** |
| Chronic pulmonary disease, N (%) | 14 (32.6) | 21 (39.6) | 10 (41.7) | 25 (44.6) | 0.868 |
| Malignancy, N (%) | 26 (60.5) | 9 (17.0) | 9 (37.5) | 17 (30.4) | 0.111 |
| Autoimmune disease, N (%) | 8 (18.6) | 8 (15.1) | 4 (16.7) | 14 (25.0) | 0.460^⸙^ |
| Primary immune deficiency, N (%) | 0 | 3 (5.7) | 1 (4.2) | 0 | 0.181^⸙^ |
| Diabetes mellitus, N (%) | 18 (41.9) | 15 (28.3) | 8 (33.3) | 21 (37.5) | 0.594 |
| Systemic corticosteroid therapy, N (%) | 32 (74.4) | 21 (39.6) | 13 (54.2) | 34 (60.7) | 0.084 |
| Prednisone equivalent dose, median (IQR) ^b^ | 27 (32) | 20 (28) | 30 (29) | 28 (50) | 0.369 |
| Prednisone >20 mg per days, N (%) | 28 (65.1) | 11 (20.8) | 9 (37.5) | 24 (42.9) | **0.044** |
| Immune status |  |  |  |  | **0.020^⸙^** |
| Apparently immunocompetent, N (%) | 5 (11.6) | 25 (47.2) | 6 (25.0) | 14 (25.0) |  |
| Mild-moderate immune suppression ^c^, N (%) | 8 (18.6) | 12 (22.6) | 6 (25.0) | 8 (14.3) |  |
| Substantial immune suppression ^d^, N (%) | 30 (69.8) | 16 (30.2) | 12 (50.0) | 34 (60.7) |  |
| **Nocardiosis** |  |  |  |  |  |
| *Nocardia* species ^e^ |  |  |  |  | **0.031^⸙^** |
| *Nocardia cyriacigeorgica*, N (%) | 11 (45.8) | 14 (53.8) | 9 (60.0) | 13 (43.3) |  |
| *Nocardia farcinica*, N (%) | 7 (29.2) | 1 (3.8) | 3 (20.0) | 8 (26.7) |  |
| *Nocardia otitidiscaviarum*, N (%) | 3 (12.5) | 0 | 1 (6.7) | 3 (10.0) |  |
| *Nocardia brasiliensis*, N (%) | 0 | 5 (19.2) | 1 (6.7) | 0 |  |
| Miscellaneous *Nocardia* spp., N (%) | 3 (12.5) | 6 (23.1) | 1 (6.7) | 6 (20.0) |  |
| Nocardiosis syndrome |  |  |  |  | **<0.001^⸙^** |
| Solitary lymphocutaneous, N (%) | 0 | 14 (26.4) | 6 (25.0) | 3 (5.4) |  |
| Osteomyelitis, N (%) | 1 (2.3) | 1 (1.9) | 1 (4.2) | 0 |  |
| Solitary pulmonary, N (%) | 24 (55.8) | 32 (60.4) | 13 (54.2) | 28 (50.0) |  |
| Disseminated, no CNS involvement, N (%) | 3 (7.0) | 3 (5.7) | 2 (8.3) | 10 (17.9) |  |
| Disseminated, CNS involvement, N (%) | 15 (34.9) | 3 (5.7) | 2 (8.3) | 15 (26.8) |  |
| Oxygen supplementation during hospitalization |  |  |  |  | 0.713^⸙^ |
| No need, N (%) | 10 (23.8) | 32 (71.1) | 16 (80.0) | 35 (63.6) |  |
| Nasal canula, N (%) | 10 (23.8) | 6 (13.3) | 3 (15.0) | 11 (20.0) |  |
| High-flow nasal canula, N (%) | 4 (9.5) | 1 (2.2) | 0 | 4 (7.3) |  |
| Non-invasive ventilation, N (%) | 2 (4.8) | 0 | 0 | 0 |  |
| Mechanical ventilation, N (%) | 16 (38.1) | 6 (13.3) | 1 (5.0) | 5 (9.1) |  |
| C-reactive protein at presentation (mg/dL), median (IQR) | 12.6 (22.0) | 5.9 (16.0) | 8.1 (12.0) | 9.1 (18.0) | 0.347 |
| **Treatment** |  |  |  |  |  |
| SXT-based regimen, N (%) | 37 (86.0) | 44 (83.0) | 19 (79.2) | 48 (85.7) | 0.748^⸙^ |
| Combination treatment, N (%) | 33 (76.7) | 28 (52.8) | 16 (66.7) | 46 (82.1) | **0.005** |
| Combination treatment duration (weeks), median (IQR) | 2.0 (5.0) | 5.5 (7.8) | 6.0 (12.5) | 13.5 (26.0) | **<0.001** |
| IV therapy duration (weeks), median (IQR) | 1.8 (2.9) | 1.0 (6.4) | 5.5 (17.0) | 5.5 (21.6) | **<0.001** |
| Antibiotic-related side effects, N (%) | 16 (37.2) | 20 (37.7) | 10 (41.7) | 19 (33.9) | 0.794 |
| Renal toxicity, N (%) | 6 (15.0) | 5 (10.0) | 3 (15.8) | 8 (14.3) | 0.719^⸙^ |
| Bone marrow toxicity, N (%) | 9 (22.5) | 9 (18.0) | 3 (14.3) | 7 (12.7) | 0.801^⸙^ |
| *C. difficile* infection, N (%) | 1 (2.5) | 2 (4.0) | 1 (5.0) | 3 (5.4) | 0.999^⸙^ |
| Allergic reaction, N (%) | 0 | 6 (12.0) | 1 (5.0) | 5 (9.1) | 0.779^⸙^ |
| Surgical intervention, N (%) | 4 (9.8) | 11 (21.6) | 5 (22.7) | 14 (25.5) | 0.891 |
| **Outcomes at one year from diagnosis** |  |  |  |  |  |
| Rehospitalization, N (%) | NA | 24 (45.3) | 11 (45.8) | 29 (51.8) | 0.770 |
| Nocardiosis relapse, N (%) | NA | 0 | 0 | 2 (3.6) | 0.662^⸙^ |
| All-cause mortality, N (%) | NA | 6 (11.3) | 5 (20.8) | 9 (16.1) | 0.505^⸙^ |

^a^ Calculated for the three treatment groups using Chi-square test or Fisher’s exact test (^⸙^) and one-way ANOVA for categorical and continuous variables, respectively; ^b^ Average daily prednisone dosage (in milligrams), during the 90 days prior to presentation. Whenever dexamethasone was used, dosage was converted to the equivalent prednisone dosage; ^c^ Individuals with active malignancy, primary immune deficiency, autoimmune diseases, and chronic corticosteroid therapy (≥5 and <20 mg per day); ^d^ Solid organ transplant recipients, hematopoietic stem cell transplant recipients, and individuals with acquired immunodeficiency syndrome (AIDS) or on chronic corticosteroid therapy (≥20 mg per day); ^e^ Identification to the species level was available for 95/176 (54%) of the cohort. IV = intravenous; NA = not applicable; SXT = sulfamethoxazole-trimethoprim.

# **Table S8. Clinical characteristics and outcomes of individuals diagnosed with nocardiosis, according to treatment duration range: sensitivity analysis using a cutoff of 180 days**

|  | **Died within**  **90 days**  **N = 43 (24%)** | **Treatment duration** | | ***p* ^a^** |
| --- | --- | --- | --- | --- |
|  |  | **≤180 days**  **N = 77 (58%)** | **>180 days**  **N = 56 (42%)** |  |
| **Demographics and comorbidities** |  |  |  |  |
| Age at diagnosis, median (IQR) | 67 (21) | 61 (25) | 61 (22) | 0.868 |
| Female gender, N (%) | 14 (32.6) | 34 (44.2) | 26 (46.4) | 0.795 |
| Charlson score, median (IQR) | 4 (4) | 2.0 (3.0) | 2.0 (3.0) | 0.239 |
| Solid organ transplant recipient, N (%) | 4 (9.3) | 10 (13.0) | 10 (17.9) | 0.438 |
| Hematopoietic stem cell transplant recipient, N (%) | 5 (11.6) | 5 (6.5) | 9 (16.1) | 0.076 |
| Chronic pulmonary disease, N (%) | 14 (32.6) | 31 (40.3) | 25 (44.6) | 0.613 |
| Malignancy, N (%) | 26 (60.5) | 18 (23.4) | 17 (30.4) | 0.367 |
| Autoimmune disease, N (%) | 8 (18.6) | 12 (15.6) | 14 (25.0) | 0.176 |
| Primary immune deficiency, N (%) | 0 | 4 (5.2) | 0 | 0.138^⸙^ |
| Diabetes mellitus, N (%) | 18 (41.9) | 23 (29.9) | 21 (37.5) | 0.356 |
| Systemic corticosteroid therapy, N (%) | 32 (74.4) | 34 (44.2) | 34 (60.7) | 0.059 |
| Prednisone equivalent dose, median (IQR) ^b^ | 27 (32) | 20 (31) | 28 (50) | 0.262 |
| Prednisone >20 mg per days, N (%) | 28 (65.1) | 20 (26.0) | 24 (42.9) | **0.041** |
| Immune status |  |  |  | **0.021** |
| Apparently immunocompetent, N (%) | 5 (11.6) | 31 (40.3) | 14 (25.0) |  |
| Mild-moderate immune suppression ^c^, N (%) | 8 (18.6) | 18 (23.4) | 8 (14.3) |  |
| Substantial immune suppression ^d^, N (%) | 30 (69.8) | 28 (36.4) | 34 (60.7) |  |
| **Nocardiosis** |  |  |  |  |
| *Nocardia* species ^e^ |  |  |  | **0.038^⸙^** |
| *Nocardia cyriacigeorgica*, N (%) | 11 (45.8) | 23 (56.1) | 13 (43.3) |  |
| *Nocardia farcinica*, N (%) | 7 (29.2) | 4 (9.8) | 8 (26.7) |  |
| *Nocardia otitidiscaviarum*, N (%) | 3 (12.5) | 1 (2.4) | 3 (10.0) |  |
| *Nocardia brasiliensis*, N (%) | 0 | 6 (14.6) | 0 |  |
| Miscellaneous *Nocardia* spp., N (%) | 3 (12.5) | 7 (17.1) | 6 (20.0) |  |
| Nocardiosis syndrome |  |  |  | **<0.001^⸙^** |
| Solitary lymphocutaneous, N (%) | 0 | 20 (26.0) | 3 (5.4) |  |
| Osteomyelitis, N (%) | 1 (2.3) | 2 (2.6) | 0 |  |
| Solitary pulmonary, N (%) | 24 (55.8) | 45 (58.4) | 28 (50.0) |  |
| Disseminated, no CNS involvement, N (%) | 3 (7.0) | 5 (6.5) | 10 (17.9) |  |
| Disseminated, CNS involvement, N (%) | 15 (34.9) | 5 (6.5) | 15 (26.8) |  |
| Oxygen supplementation during hospitalization |  |  |  | 0.323^⸙^ |
| No need, N (%) | 10 (23.8) | 48 (73.8) | 35 (63.6) |  |
| Nasal canula, N (%) | 10 (23.8) | 9 (13.8) | 11 (20.0) |  |
| High-flow nasal canula, N (%) | 4 (9.5) | 1 (1.5) | 4 (7.3) |  |
| Non-invasive ventilation, N (%) | 2 (4.8) | 0 | 0 |  |
| Mechanical ventilation, N (%) | 16 (38.1) | 7 (10.8) | 5 (9.1) |  |
| C-reactive protein at presentation (mg/dL), median (IQR) | 12.6 (22.0) | 6.7 (15.0) | 9.1 (18.0) | 0.226 |
| **Treatment** |  |  |  |  |
| SXT-based regimen, N (%) | 37 (86.0) | 63 (81.8) | 48 (85.7) | 0.550 |
| Combination treatment, N (%) | 33 (76.7) | 44 (57.1) | 46 (82.1) | **0.002** |
| Combination treatment duration (weeks), median (IQR) | 2.0 (5.0) | 6.0 (9.5) | 13.5 (26.0) | **<0.001** |
| IV therapy duration (weeks), median (IQR) | 1.8 (2.9) | 1.0 (9.4) | 5.5 (21.6) | **0.003** |
| Antibiotic-related side effects, N (%) | 16 (37.2) | 30 (39.0) | 19 (33.9) | 0.552 |
| Renal toxicity, N (%) | 6 (15.0) | 8 (11.6) | 8 (14.3) | 0.654 |
| Bone marrow toxicity, N (%) | 9 (22.5) | 12 (16.9) | 7 (12.7) | 0.516 |
| *C. difficile* infection, N (%) | 1 (2.5) | 3 (4.3) | 3 (5.4) | 0.999^⸙^ |
| Allergic reaction, N (%) | 0 | 7 (10.0) | 5 (9.1) | 0.864 |
| Surgical intervention, N (%) | 4 (9.8) | 16 (21.9) | 14 (25.5) | 0.640 |
| **Outcomes at one year from diagnosis** |  |  |  |  |
| Rehospitalization, N (%) | NA | 35 (45.5) | 29 (51.8) | 0.471 |
| Nocardiosis relapse, N (%) | NA | 0 | 2 (3.6) | 0.175^⸙^ |
| All-cause mortality, N (%) | NA | 11 (14.3) | 9 (16.1) | 0.776 |

^a^ Calculated for the three treatment groups using Chi-square test or Fisher’s exact test (^⸙^) and Mann-Whitney test for categorical and continuous variables, respectively; ^b^ Average daily prednisone dosage (in milligrams), during the 90 days prior to presentation. Whenever dexamethasone was used, dosage was converted to the equivalent prednisone dosage; ^c^ Individuals with active malignancy, primary immune deficiency, autoimmune diseases, and chronic corticosteroid therapy (≥5 and <20 mg per day); ^d^ Solid organ transplant recipients, hematopoietic stem cell transplant recipients, and individuals with acquired immunodeficiency syndrome (AIDS) or on chronic corticosteroid therapy (≥20 mg per day); ^e^ Identification to the species level was available for 95/176 (54%) of the cohort. IV = intravenous; NA = not applicable; SXT = sulfamethoxazole-trimethoprim.
